# Confocal Raman Spectral Imaging Study of DAPT, a γ-secretase Inhibitor, Induced Physiological and Biochemical Reponses in Osteosarcoma Cells

**DOI:** 10.7150/ijms.43506

**Published:** 2020-02-10

**Authors:** Jie Li, Rui Wang, Jie Qin, Haishan Zeng, Kaige Wang, Qingli He, Difan Wang, Shuang Wang

**Affiliations:** 1 Institute of Photonics and Photon-Technology, Northwest University, Xi'an, Shaanxi 710069, China; 2 Department of Orthopedics, The Second Affiliated Hospital of Xi'an Jiaotong University, Xi'an, Shaanxi 710004, China; 3 Imaging Unit - Integrative Oncology Department, BC Cancer Research Center, Vancouver, BC, V5Z1L3, Canada; 4 Department of Physics, Northwest University, Xi'an, Shaanxi 710069, China; 5 School of Life, Xidian University, Xi'an, Shaanxi 710071, China

**Keywords:** Confocal Raman microspectral imaging, Osteosarcoma cells, DAPT, Drug Responses, Cellular Heterogeneity

## Abstract

Confocal Raman microspectral imaging was adopted to elucidate the cellular drug responses of osteosarcoma cells (OC) to N-[N-(3, 5-difluorophenyl acetyl)-L-alanyl]-sphenylglycine butyl ester (DAPT), a γ-secretase inhibitor, by identifying the drug induced subcellular compositional and structural changes.

**Methods**: Spectral information were acquired from cultured osteosarcoma cells treated with 0 (Untreated Group, UT), 10 (10 μM DAPT treated, 10T), 20 μM (20 μM DAPT treated, 20T) DAPT for 24 hours. A one-way ANOVA and Tukey's honest significant difference (HSD) post hoc multiple test were sequentially applied to address spectral features among three groups. Multivariate algorithms such as K-means clustering analysis (KCA) and Principal component analysis (PCA) were used to highlight the structural and compositional differences, while, univariate imaging was applied to illustrate the distribution pattern of certain cellular components after drug treatment.

**Results**: Major biochemical changes in DAPT-induced apoptosis came from changes in the content and structure of proteins, lipids, and nucleic acids. By adopted multivariate algorithms, the drug induced cellular changes was identified by the morphology and spectral characteristics between untreated cells and treated cells, testified that DAPT mainly acted in the nuclear region. With the increase of the drug concentration, the content of main subcellular compositions, such nucleic acid, protein, and lipid decreased. In an addition, DAPT-induced nuclear fragmentation and apoptosis was depicted by the univariate Raman image of major cellular components (nucleic acids, proteins and lipids).

**Conclusions**: The achieved Raman spectral and imaging results illustrated detailed DAPT-induced subcellular compositional and structural variations as a function of drug dose. Such observations can not only explain drug therapeutic mechanisms of OC DAPT treatment, and also provide new insights for accessing the medicine curative efficacy and predicting prognosis.

## Introduction

Osteogenic sarcoma, also referred as osteosarcoma (OS), is the most common primary malignant tumor originating from primitive mesenchymal cells 1,2. OS is characterized by a high degree of metastasis 3-5, and it affects patients of all ages, but showing a substantially higher incidence in children and early adulthood, which can lead to childhood and adolescent disability 6. Platinum-based anticancer drugs including cisplatin, carboplatin, and oxaliplatin are among the most potent and widely used chemotherapeutic agents in the clinic 6. However, the effect of platinum-based chemotherapy increases with dose, but high doses of platinum-based drugs are accompanied by severe side effects during treatment 7. Multi-chemotherapy is also an effective treatment of osteosarcoma, but more than 90% of patients with osteosarcoma die of lung metastases before multi-chemotherapy 8. Therefore, there is an urgent need to develop new and effective drugs that not only kill primary tumors with lowest side effects but also inhibit metastasis as early as possible 9.

The N-[N-(3,5-difluorophenyl acetyl)-L-alanyl]-sphenylglycine butyl ester (DAPT) 10 is a γ-secretase inhibitor that inhibits the third-order digestion of the γ-secretase substrate Notch. DAPT reduces the release of intracellular Notch receptor and affects the cell signaling and differentiation processes regulated by the Notch signaling pathway 10, 11. This is an evolutionarily conserved mechanism involved in cell proliferation, differentiation, and apoptosis 12-14. Recently, Liu *et al*. 10 proved that DAPT can significantly inhibit the growth and proliferation of SHG-44 cells *via* a 3-4,5-dimethylthiazol-2,5 diphenyl tetrazolium bromide (MTT) assay, and the results of flow cytometry analysis showed that DAPT is an effective drug for the treatment of glioma. Treatment with selective DAPT can prevent the epithelial-mesenchymal transition (EMT) and inhibit the *in vitro* migration of osteosarcoma cells 15.

Raman spectroscopy is a non-destructive technique that provides label-free molecular information on biological samples. The positions, intensities, and line-widths of various spectral bands can be used to probe the primary, secondary, tertiary, and quaternary structures of biological molecules 16. Confocal Raman microspectral imaging (CRMI) is a Raman based spectral imaging technique that describes the composition and distribution pattern of biochemicals in cells (e.g. amino acids and proteins, lipids, and nucleic acids) without alteration of cellular functions 17-19. It has been widely used to study cellular events such as cell growth and death induced by stimuli, drugs, or toxins 17,20-22 as well as cellular physiologies at different time-points in the cell cycle 23. Combined with univariate and multivariate analysis, the compositional and structural information provided by CRMI can quantitatively and temporally describe the dose-related cell responses to drugs or other treatment modalities. Such information can illustrate the therapeutic mechanism of new treatment methods for cancer or other major diseases.

Based on our previous biomedical study 24, we used CRMI with our previously reported single-cell spectral imaging protocol 25 to study drug responses of OS cells to different DAPT concentrations (10, 20 μM) for 24 hours. Cancer-drug interactions that induced content changes in main cellular components were depicted by the spectral contribution of their featured Raman spectral signatures. Multivariate analysis including K-means clustering (KCA) and principal component analysis (PCA) was applied to elaborate the drug response at different subcellular regions *via* intragroup analysis with spectral imaging method. DAPT induced subcellular compositional and structural variations were then analyzed *via* a pair-wised comparison among different doses. The study provides unique illustration on detail DAPT-induced cellular changes and heterogeneity as a function of drug dose. These changes can explain drug therapeutic and resistance mechanisms and provide new insights for the development of molecular-targeted medicine.

## Experimental

### Sample preparation

Murine osteosarcoma cell line K7M2 from American Type Culture Collection (ATCC) was used in this work. It was cultured in DMEM supplemented with 10% (v/v) FBS and 1% P&S in an incubator at 37°C in 5% CO_2_. The medium was changed every two days. The cells were passaged every four days. Raman spectral detection was done after 3 to 15 passages. The cells were seeded on a 2.5 cm diameter CaF_2_ substrate in a 6 cm diameter cell culture dish, and cultured in different DAPT concentrations for 24 hours. DAPT was dissolved in DMSO at a storage concentration of 50 mM and diluted to a working concentration of 10 µM (10T group) and 20 µM (20T group). The 0 µM DAPT group (Untreated control group) had the same amount of DMSO but without DAPT.

### Confocal Raman Microspectroscopy

The confocal Raman microscopy system used in this study has been described previously 25. Briefly, a fiber coupled to a 532-nm semiconductor laser was collimated into A 63× water-immersion objective lens (NA=1.0, W Plan-APOCHROMAT, Zeiss, Germany) for Raman excitation and spectral measurements. Cells seeded CaF_2_ slides were placed on a multi-axis piezo scanning stage (P-524K081, PI GmbH, Germany) for point-by-point spectral scanning. The spectrum from every scanning point was recorded by a spectrometer (UHTS300, WITec GmbH, Germany) incorporating a 600 mm^-1^ grating blazed at 500 nm and a back-illuminated deep-depletion charge coupled device camera (CCD) (Du401A-BR-DD-352, Andor Technology, UK) working at -60 ˚C. All measurements were performed at room temperature after system wavelength and spectral intensity calibration.

During experiment, the Raman spectra from UT, 10T and 20T groups were measured randomly from 20 spots inside the cell structure. A total of 12 cells were measured in all groups (UT group, 4 cells; 10T group, 4 cells; 20T group, 4 cells), 4 biological replicants were performed (UT group, 1 replicant; 10T group, 1 replicant; 20T, 2 replicants), and totally 60 Raman spectra were considered (UT group, 20 Raman spectra; 10T group, 20 Raman spectra; 20T group, 20 Raman spectra).

### Spectral data pre-processing and imaging analysis

Spectral pre-preprocessing used WITec Project 4 (WITec GmbH) following our previously protocols 25. Briefly, both the low wavenumber (600-1800 cm^-1^) and high wavenumber (2800-3100 cm^-1^) domains were intercepted after 5 order polynomial fitting and 4-point Savitzky-Golay (S-G) smoothing. A one way analysis of variance (ANOVA) was performed followed by Tukey's honest significant difference (HSD) post-hoc to determine differences in peak areas between groups with 95% confidence, and analysis and drawing in Sigmaplot software (Sigmaplot 12.5). After that, point-scanned spectral datasets were prepared for further multivariate and univariate spectral imaging using WITec Project (Ulm, Germany) software. After spectral analysis of the treated cells and control cells, KCA was used to identify and classify similar components in the cells, and the divided clusters were shown as pseudo-color image to visualize the subcellular structure. PCA was implemented by a home-made algorithm using Matlab data-processing functions (The Mathworks, Inc., United States). This procedure reduces the number of variables in the cube while preserving most of the primary information in the data set 26. Only the first two principal components (PCs) were considered when interpreting the spectral information of sub-cellular biochemical constitutions because they indicated the main spectral variances in the dataset. Univariate spectral imaging was utilized to evaluate the integrated intensity of specific peaks found within the scanning area to access the distribution of the biochemical phenotypes.

## Results

### Spectral features in the UT, 10 T, and 20 T groups

**Figure 1:**
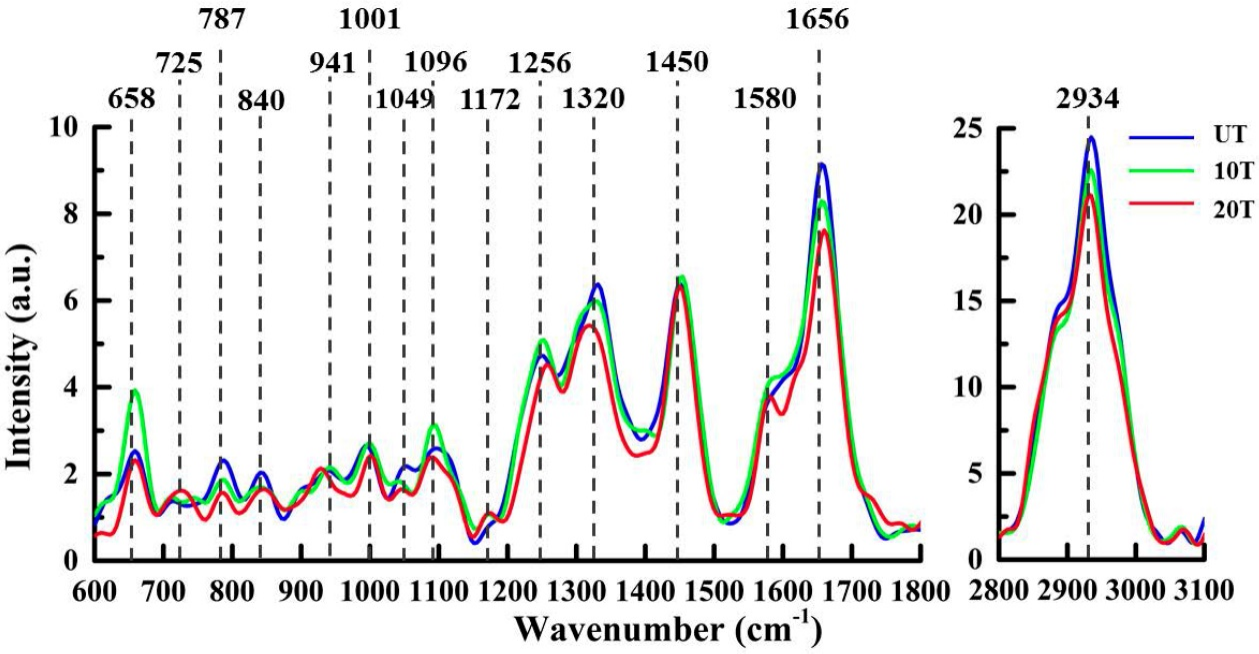
Mean Raman spectra of osteosarcoma cells in the UT, 10T and 20T groups.

A comparison among the mean pro-processed Raman spectra of UT, 10T and 20T groups is shown in Figure 1. The pro-processed and averaged spectra were further normalized by the area-under-the-curve method to minimize the effect due to sample and instrument variabilities such as sample inhomogeneity and drift in excitation light intensity 25. The spectra variations among tested groups was small and some systematic characteristics could be perceived around 658, 725, 787, 840, 941, 1001, 1049, 1096, 1172, 1256, 1320, 1450, 1580, 1656, and 2934 cm^-1^; their biochemical assignments are listed in Table 1.

Compared with the mean spectra of untreated osteosarcoma cell, both DAPT-treated groups showed a decrease in the spectral features at 787 cm^-1^ (uracil, thymine, and cytosine, DNA backbone O-P-O stretching) 27,28, 840 cm^-1^ (carbohydrate, glucose) 29, 1320 cm^-1^ (protein, C-H) 30,31, 1656 cm^-1^(protein, amide I, α-spiral) 19,32 and 2934 cm^-1^ (lipid, CH_3_) 27, associated with nucleic acid, protein, lipid, and carbohydrate content. In both 10T and 20T groups, a decrease in peak intensity, as compared to the UT group, was observed at 787 cm^-1^, which is associated with uracil, thymine, and cytosine ring breathing modes in DNA and RNA bases. The spectra also contain contributions from the O-P-O backbone of DNA that can be used as an indicator of the nucleic acid content variations during cell responses to DAPT 27,28. A shift to a lower wavenumber was observed for the peak at 1096 cm^-1^ corresponding to O-P-O stretching and indicating that DAPT can also bind to the outside of the DNA 33,34.

As the drug dose increased from 10 μM to 20 μM, not only the intensities of the protein Raman peaks showed a decreased trend, but also 1320 cm^-1^ (C-H deformation of protein) and 1256 cm^-1^ (amide III bond of proteins) bands showed obvious bands shifts (1320 to 1317 cm^-1^,1256 to 1260 cm^-1^). This might be related to protein mutation after the interaction between DAPT and cancer cells 35,36. As well, the C=C stretching vibration of unsaturated fatty acids experiences an intensity decrease. Moreover, the lipid-related Raman band vibration at 2934 cm^-1^ as well as the CH_3_ stretching vibration decreased with increasing drug concentration 27.

Compared with cells in the UT group, the peaks at 658 cm^-1^ (tyrosine and C-C twist) 29, 1001 cm^-1^ (phenylalanine) 27, and 1256 cm^-1^ (amide III, α-spiral, C=C str.) 27,37 increased in the 10T group and decreased in the 20T group. The 840 cm^-1^ (glucose), 941 cm^-1^ (C-C BK str.α-helix) 19, 1001 cm^-1^ (phenylalanine), 1256 cm^-1^ (amide III) 27,37, and 1320 cm^-1^ (C-H) peak positions were shifted to higher wavenumber; the 725 cm^-1^ (adenine) 38 peak position could also be identified. We suspected that this may be due to the high concentration (20 µM) of drugs inducing stronger interactions with proteins and nucleic acids in the cells causing protein denaturation and the appearance of free adenine in the cells.

**Figure 2:**
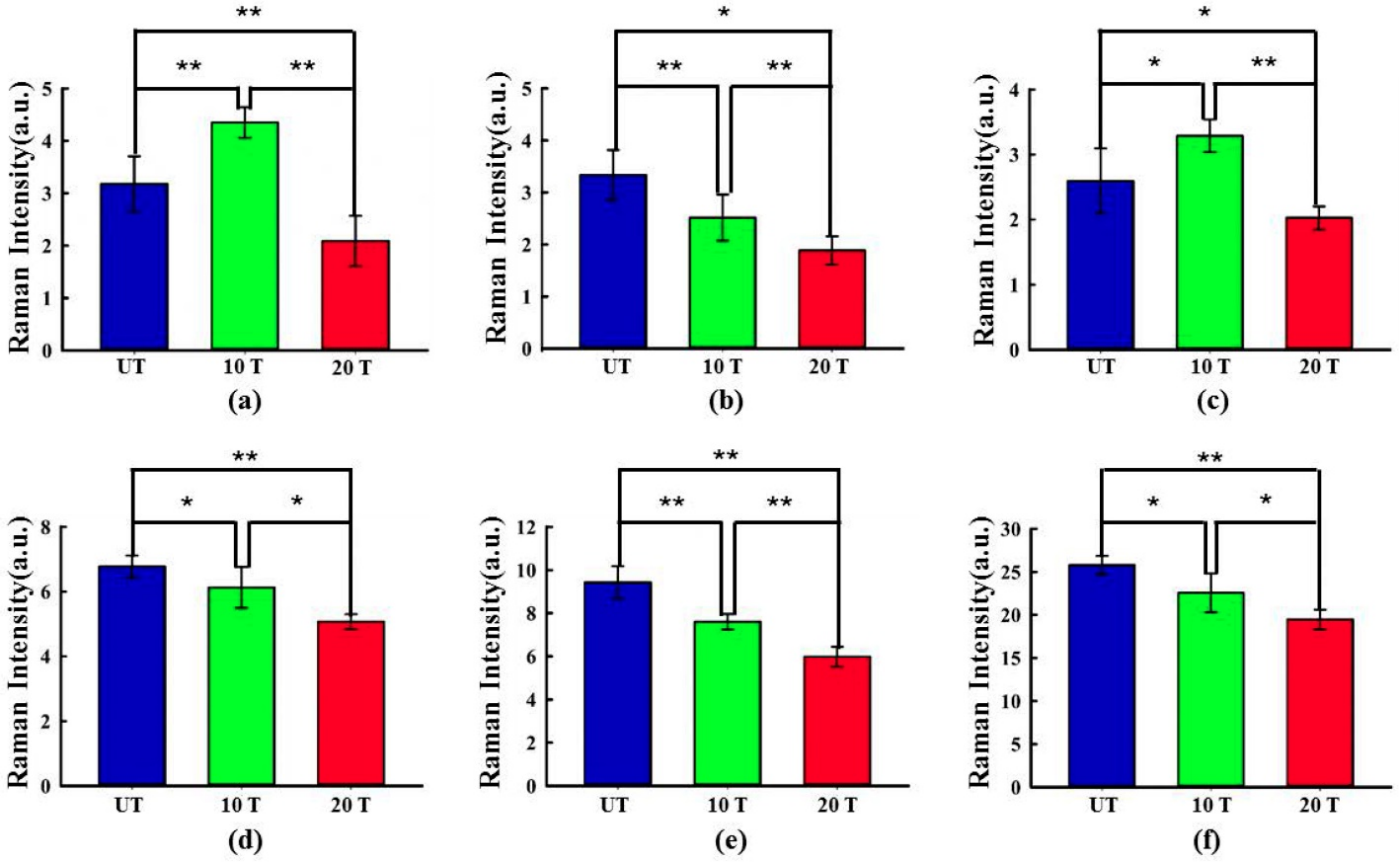
Box chart displaying the relative spectral contributions of biochemical species in sampled osteosarcoma cells in the UT group (blue), 10 UT group (green) and 20 UT group (red). Spectral contributions for each species were calculated and displayed in (a) protein (658 cm^-1^), (b) nucleic acid (787 cm^-1^), (c) nucleic acid (1096 cm^-1^), (d) protein (1320 cm^-1^), (e) protein and lipid (1656 cm^-1^), (f) lipid (2934 cm^-1^). Each species is plotted as mean ± standard deviation. Statistical significance was determined by one-way ANOVA followed by a post hoc Tukey's HSD test. Asterisks indicate levels of significance, *P < 0.05, **P < 0.01.

To analyze the spectral variations in more detail, one-way ANOVA and HSD post hoc multiple tests were applied sequentially to compare the changes in the individual spectral contribution in the UT, 10T, and 20T groups. Figure 2 shows the inter-group comparisons of relative spectral contribution among the three groups in which asterisks indicate the levels of significance, which is achieved by calculating the full width at half maximum (FWHM) for each peak. Remarkable variations can be observed in the spectral contribution of protein (658 cm^-1^), nucleic acids (787, 1096 cm^-1^), protein (1320 cm^-1^), protein and lipid (1656 cm^-1^), and lipid (2934 cm^-1^). Compared with untreated cells, protein 658 cm^-1^ showed two different variation trends attributed to tyrosine content and C-C distortion changes (Figure 2(a)) 29. The decrease in the spectral contribution of the proteins at 1320 cm^-1^ (C-H) and 1656 cm^-1^ (amide I, α-spiral) may be due to changes in the secondary structure of the cells' protein after drug treatment 39 (Figure 2(d) and (e)). The change in nucleic acid levels are illustrated in Figure 2(b) and (c). The spectral contribution of the nucleic acid (1096 cm^-1^; PO_2_ symmetric stretching) increased in the 10T group and gradually decreased in the 20T group. The strength of the nucleic acid peak at 787 cm^-1^ decreased as the concentration of the drug increases. This may be due to DNA damage and DNA-repair pathway inhibition in DAPT-treated cells 39. The sharp drop in lipid spectral contributions may be related to vibrational mode change of the CH_3_ stretching band 27,40 as shown in Figure 2(f).

### Spectral imaging by K-means clustering analysis

**Figure 3:**
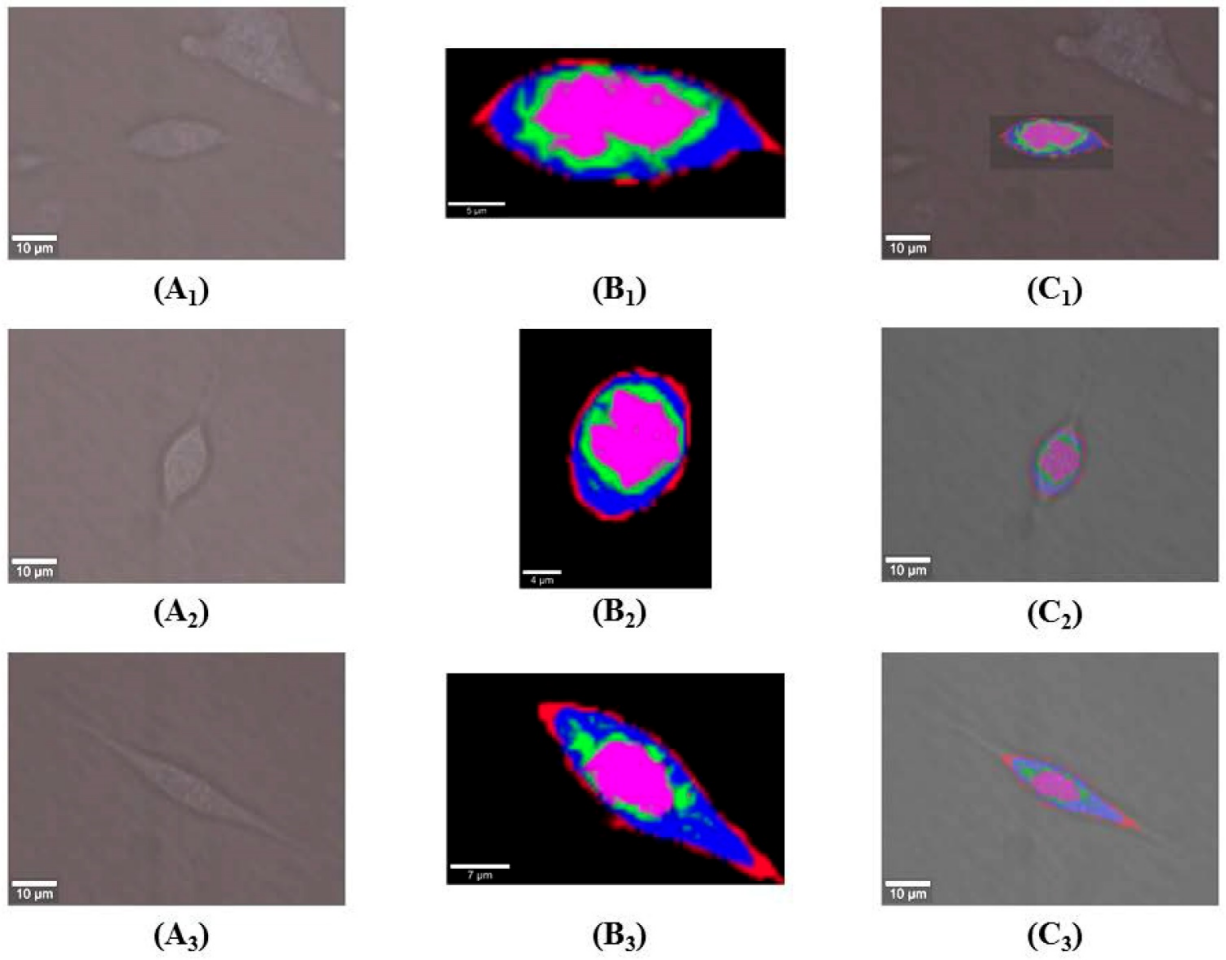
KCA pseudo-color image of Raman dataset from osteosarcoma cells of UT, 10T and 20T groups. Images (A_1_-A_3_) show white light micrographs of osteosarcoma cells in the UT, 10T and 20T groups; images (B_1_-B_3_) show the roots of a pseudo-hierarchical clustering tree of osteosarcoma cells in the UT, 10T and 20T groups; image (C_1_-C_3_) displays the spatially transformed image between (A_1_-A_3_) and (B_1_-B_3_).

KCA was adopted after spectral analysis to visualize DAPT-induced sub-cellular structural and compositional changes. Figure 3(A_1_-A_3_) shows white-light microscopic images of live osteosarcoma cells in UT, 10T, and 20T groups. KCA was further performed on each of the pro-processed data sets from 600 to 3100 cm^-1^. Raman imaging datasets from UT, 10T, and 20T groups were acquired by 1 s integration times from a 30×15 μm^2^ area containing 60×30 pixels, a 20×27 μm^2^ area containing 55×60 pixels, and a 40×25 μm^2^ area containing 80×50 pixels respectively. Pseudo-hierarchical cluster trees were generated by classifying the spectral datasets into four sub-clusters. These cluster trees clearly identified the cell membrane (red) and regions associated with a high concentration of cellular organelles (green), nucleus (purple), and cytoplasm (blue) as shown in Figure 3(B_1_-B_3_). In addition, the transformation of the root spectral image into its corresponding microscopic image reveals the morphological features corresponding to the spectral image of the cells used (Figure 3(C_1_-C_3_)).

**Figure 4:**
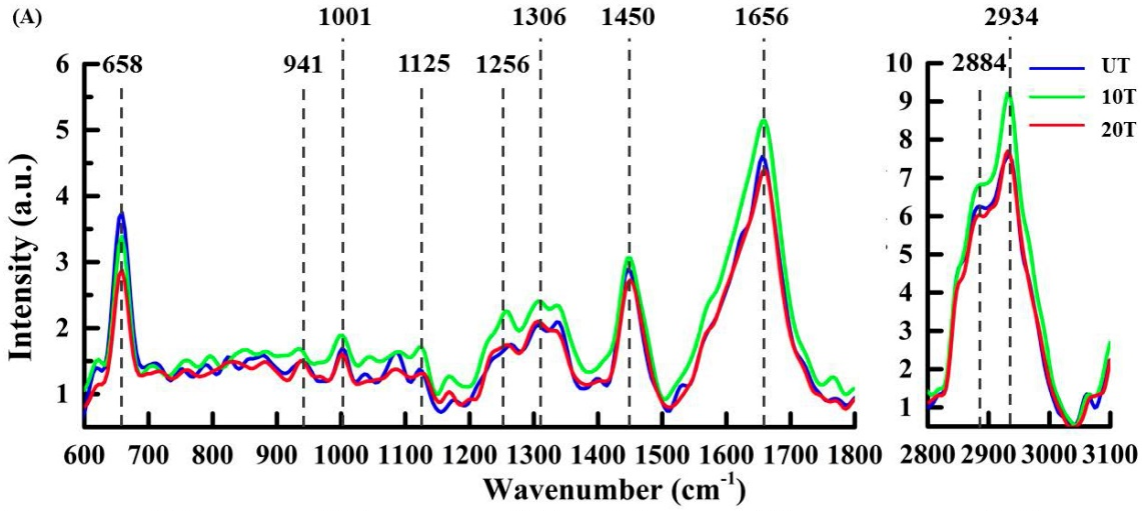

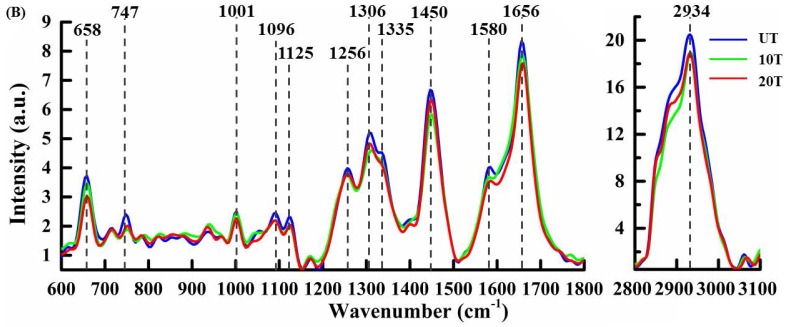

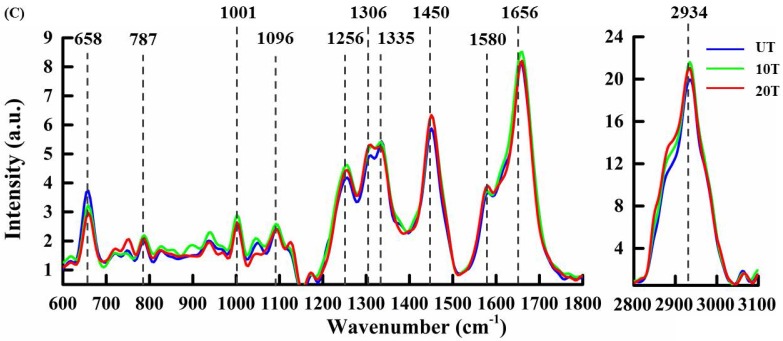

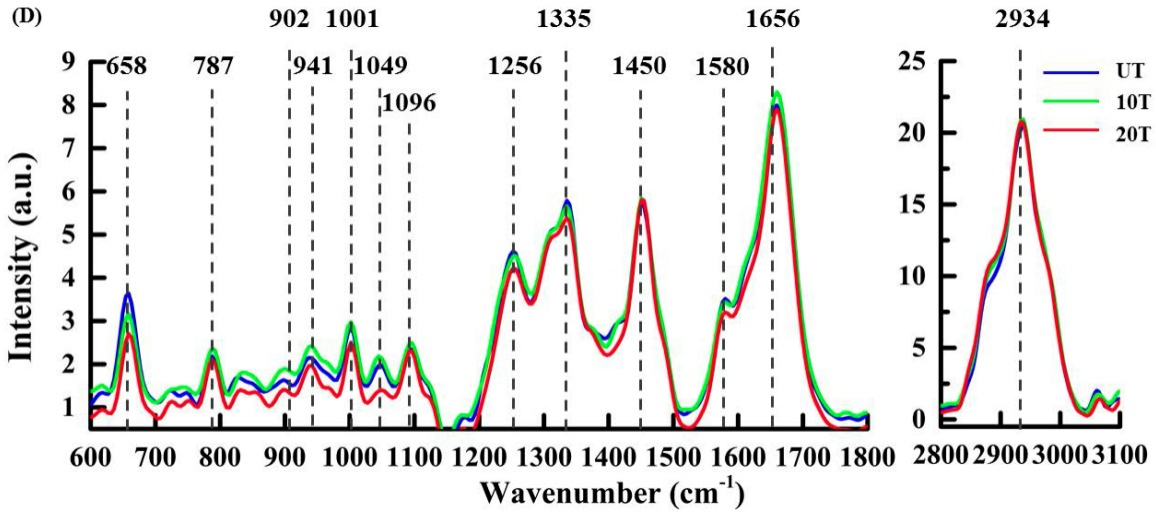
Mean baseline corrected spectra extracted from individual clusters annotated to an intracellular region in cluster Raman image: (A) cell membrane; (B) Cytoplasm; (C) Organelle; and (D) Nucleus.

After cluster separation, the mean spectra from the same sub-cluster were pairwise compared among three groups for a detailed understanding of DAPT caused by compositional variations in the same sub-cellular structures (Figure 4). In all figures, the mean spectra from UT, 10T, and 20T groups were marked with blue, green and red color, respectively. Figure 4A illustrates the classified mean spectra from the cell membrane region, which were marked red in all three groups in Figure 3B. Peak intensities of 1306 cm^-1^ (CH_2_ twist) 19, 2884 cm^-1^ (CH_3_) 40, and 2934 cm^-1^ (CH_3_) were increased to varying degrees versus control UT groups. The strength of the 1001 cm^-1^ (phenylalanine), 1125 cm^-1^ (C-N, C-C str) 29,32, 1450 cm^-1^ (CH) 27,32, and 1656 cm^-1^ (amide I, α-spiral, C=C str.) increased in the 10T group and decreased in the 20T group. After 10 μM DAPT treatment, the peak intensities of protein (1256 cm^-1^ (amide III) and 1656 cm^-1^ (amide I, α-spiral and C=C str.) increased significantly. We speculated that this might be due to changes in the secondary structure of the protein 39. The protein peak intensity at 658 cm^-1^ decreases with increasing drug concentration from tyrosine and C-C distortion 29.

Figure 4B shows the mean Raman spectra from the cytoplasm—these peaks are labeled blue in each group (Figure 3B) and exhibited some slight but evidently DAPT-induced spectral changes. The peak intensity of most sub-cellular substances in the cytoplasm are reduced after DAPT-cell interactions such as proteins at 658 cm^-1^ (tyrosine and C-C twist), 747 cm^-1^(Tyr)^28^, 1001 cm^-1^ (phenylalanine), 1125 cm^-1^ (C-N, C-C stretching), 1256 cm^-1^ (amide III), 1335 cm^-1^ (CH deformation) 30, 1450 cm^-1^(CH_2_ deformation), and 1656 cm^-1^ (amide I, α-spiral) as well as nucleic acids at 1096 cm^-1^ (PO_2_ symmetric stretching), 1335 cm^-1^ (adenine and guanine), and 1580 cm^-1^ (adenine and guanine) 41. Lipids were seen at 1125 cm^-1^ (C-C), 1306 cm^-1^ (CH_2_ twist), 1450 cm^-1^ (CH deformation), 1656 cm^-1^(C=C stretching), and 2934 cm^-1^ (CH_3_). These consistent changes in all peaks are likely because the DAPT inhibitors decreased cell viability with a reduction in most of the cytoplasmic components.

The spectral changes of the organelle (Figure 4C) are labeled green in all three groups displayed in Figure 3B. These represent the Raman bands at 1306 cm^-1^ (CH_2_ twist), 1450 cm^-1^ (C-H deformation), and 2934 cm^-1^ (CH_3_ stretching) in the membrane lipids of the organelles. A slight increase in the lipid content after DAPT treatment could be seen *via* lipid peak intensities. Compared with the UT group, the spectral intensity of proteins (1001, 1256, 1450, and 1656 cm^-1^) and nucleic acid (787, 1096, 1335, and 1580 cm^-1^) bands showed an increasing trend in both 10T and 20T groups. Whereas, the intensity of the peak at 658 cm^-1^(tyrosine and C-C twist) decreases. This may be due to the reduction of tyrosine content in organelles and the change in C-C distortion caused by the interactions of DAPT with cells 29.

The mean spectra from the nuclear clusters of treated cells in both 10T and 20T groups are exhibited in Figure 4D and compared to the UT group. The decrease in nucleic acid concentration after high-dose DAPT treatment was seen by reduced peak intensity at 787 cm^-1^ (uracil, cytosine and thymine, DNA backbone O-P-O stretching), 902 cm^-1^ (backbone) 42, 1049 cm^-1^ (RNA OPO stretching) 40, 1096 cm^-1^ (DNA backbone O-P-O stretching), 1335 cm^-1^ (adenine and guanine), and 1580 cm^-1^ (adenine and guanine). This is probably due to high concentrations of DAPT inhibitors affecting DNA replication 28,34,35,39; DNA replication is affected by destruction of DNA base pairs 35. Subtle differences in protein conformation were also observed at 658 cm^-1^ (tyrosine and C-C twist), 941 cm^-1^ (C-C BK str.α-helix), 1001 cm^-1^ (phenylalanine), 1256 cm^-1^ (amide III), and 1656 cm^-1^ (amide I) at lower DAPT concentrations. There were no significant changes in proteins (1450 cm^-1^) and lipids (2934 cm^-1^) in the nucleus.

### Spectral variations analysis by Principal Component Analysis

**Figure 5:**
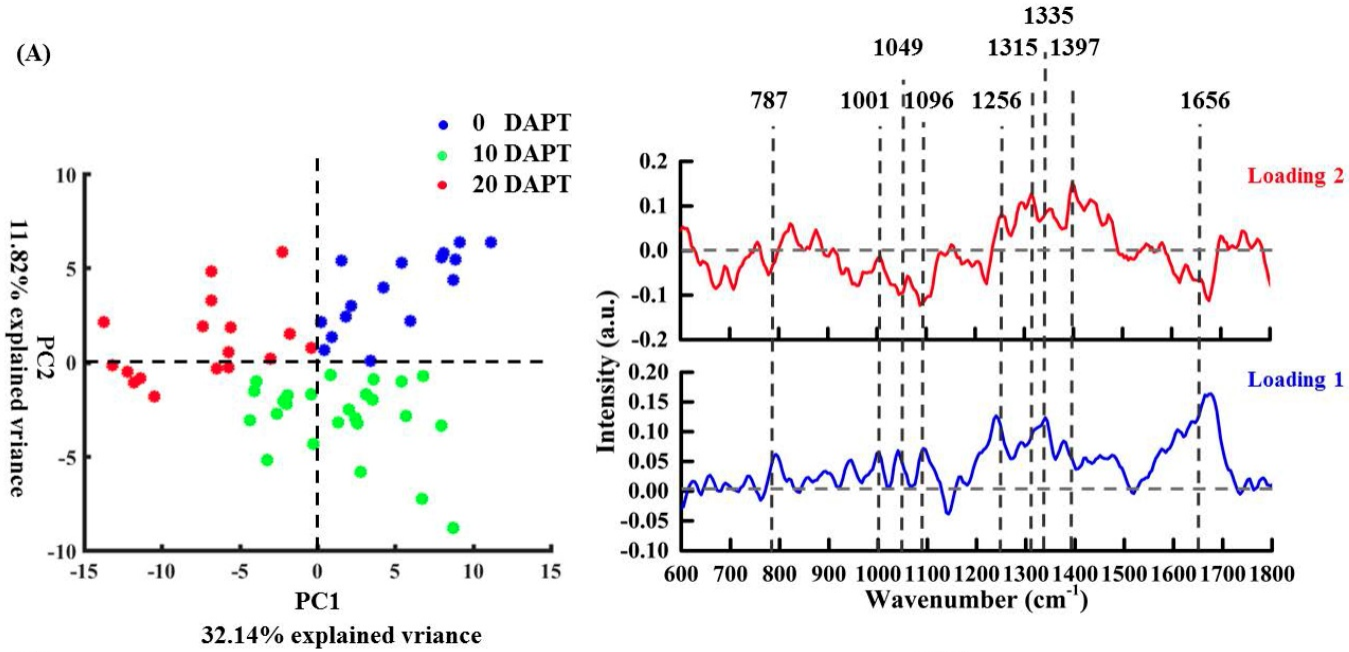

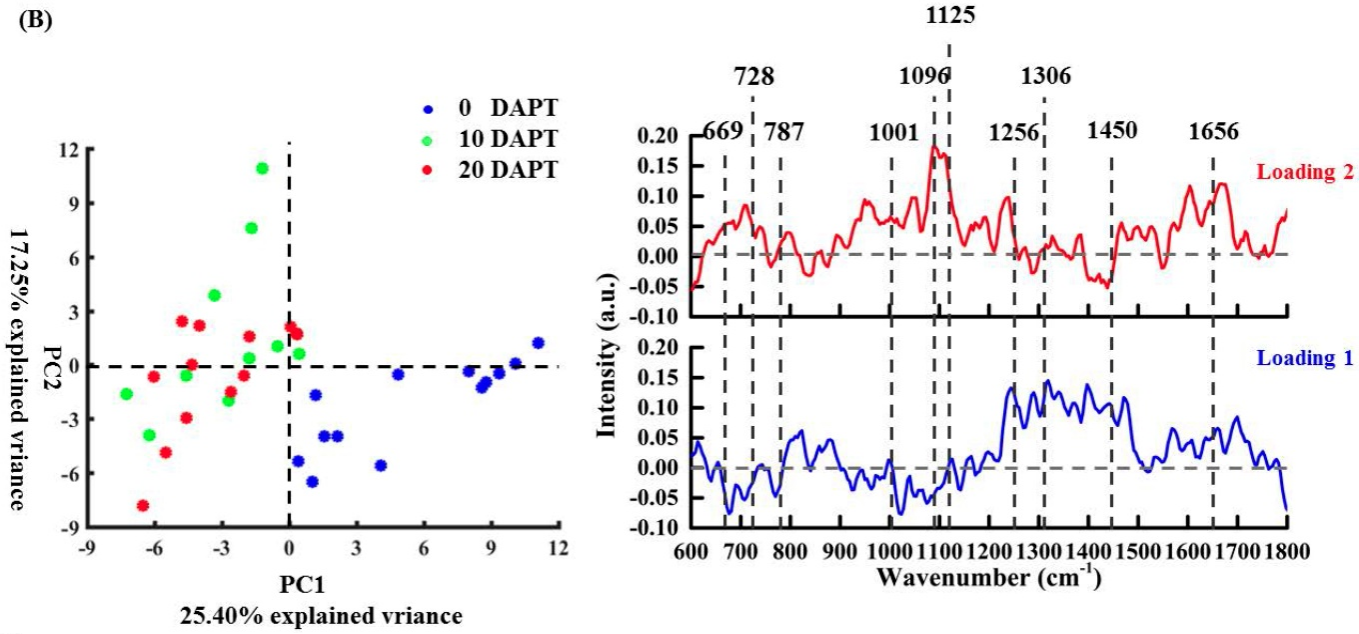

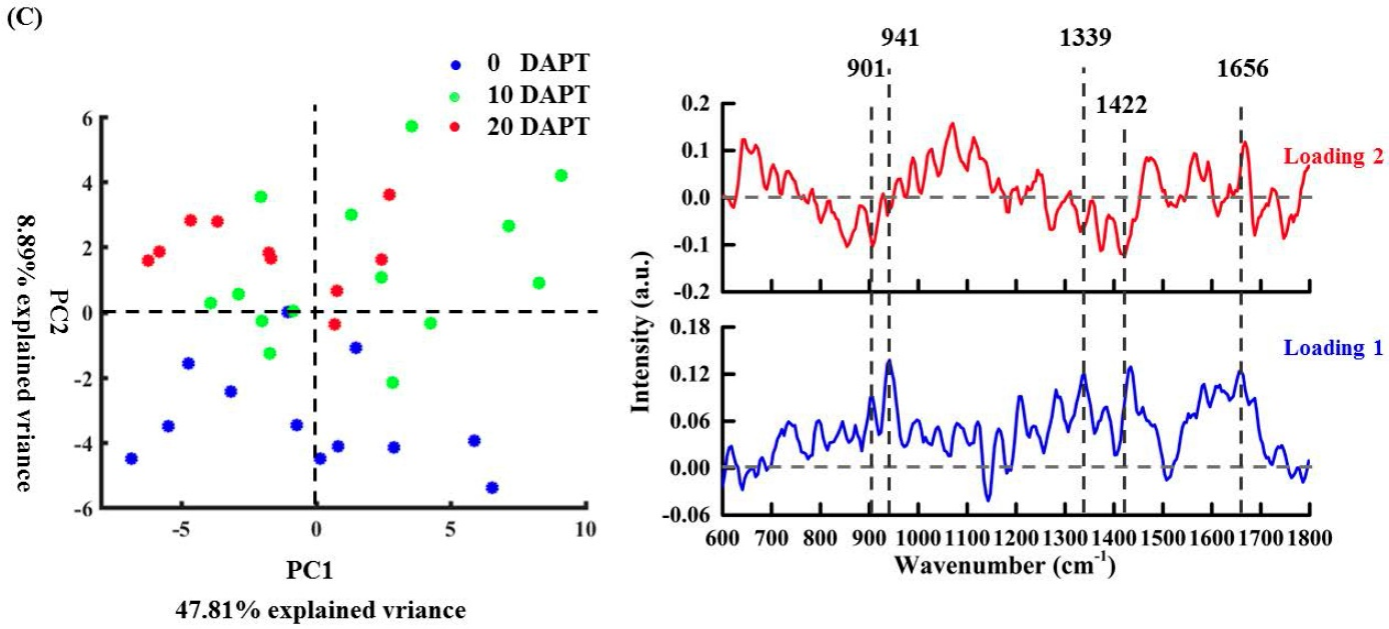

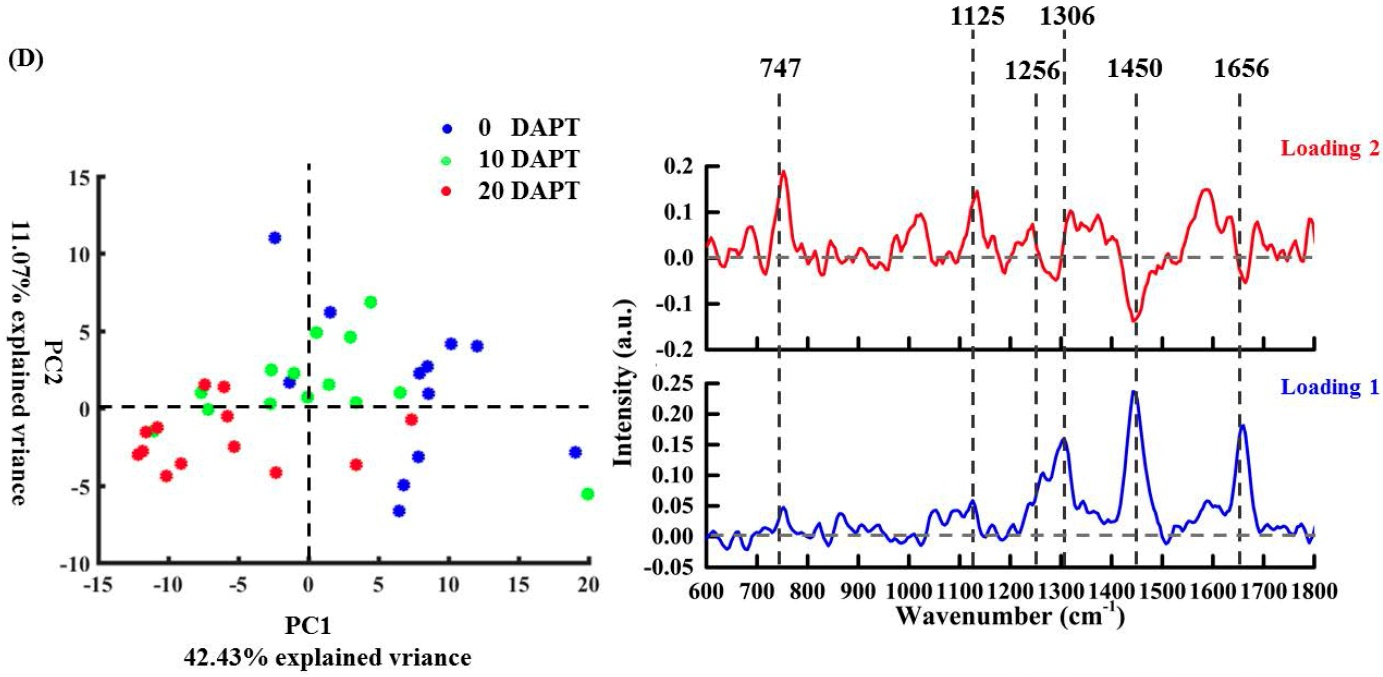

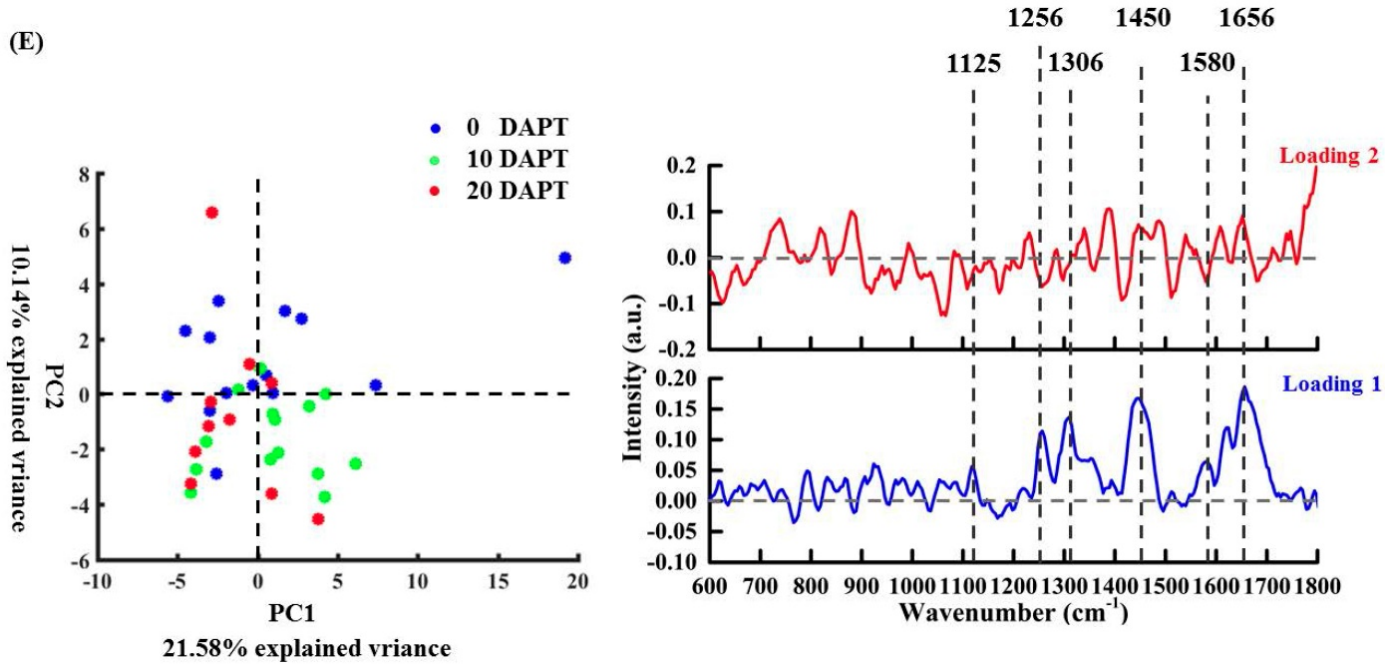
PCA of osteosarcoma cell localization for untreated (UT group) and treated (10T group, 20T group) and the corresponding loadings of PC1 and PC2 (A). Figures (B-E) shows the nuclear, organelle, cytoplasmic and cell membrane corresponding PCA for untreated (UT group) and treated (10T group, 20T group) and the corresponding loading of PC1 and PC2.

After the subcellular features were identified, PCA was adopted to facilitate a more detailed analysis on the spectral variations after DAPT treatment. Figure 5 shows both the loading score and the spectral plots of osteosarcoma cells *via* PC1 vs. PC2 from the spectra of UT, 10 T, and 20 T groups. The loading spectra are presented with both positive and negative peaks, corresponding to increased or decreased spectral contributions of specific molecular components. All loading spectra were offset for clarity with the dotted line indicating the zero point in each point.

Figure 5A shows an obvious spectral separation between untreated cells and the 20 μM DAPT-treated cells according to the scatter plot of PC1. The spectral loading of PC1 is dominated by positive features at 787 cm^-1^ (uracil, thymine, and cytosine, DNA backbone O-P-O stretching), 1001 cm^-1^ (phenylalanine), 1049 cm^-1^ (RNA OPO stretching), 1096 cm^-1^ (DNA backbone O-P-O stretching), 1335 cm^-1^ (CH deformation), and 1656 cm^-1^ (amide I, α-spiral and C=C str.). These correspond to nucleic acids, proteins, and lipids in untreated cells. These features indicate a decrease in all cellular features after high-dose DAPT. The corresponding loading of PC2 can differentiate the untreated and 10 μM DAPT-treated cells. PC2 is dominated on the positive side by features related to proteins (1256 cm^-1^, 1315 cm^-1^ (CH_2_ deformation) 40, 1397 cm^-1^ (CH_2_ rocking) 40), and nucleic acids on the negative side (787 cm^-1^, 1049 cm^-1^, 1096 cm^-1^).

To create a detailed understanding of the drug effects on each subcellular region, pairwise PCA of the nucleus, organelle, cytoplasm, and cell membrane was performed independently in Figure 5B-E. As shown in Figure 5B, data on the nucleus is obvious and illustrates that the DAPT treatment exerts strong effects on this region versus other cellular components. These findings suggest that the nucleus have a crucial role in the DAPT-induced cellular response 6,43,44. The spectral loading of PC1 has negative peaks at 669 cm^-1^ (thymine and guanine) 36, 728 cm^-1^ (adenine) 36, 787 cm^-1^ (cytosine and thymine, DNA backbone O-P-O stretching), and 1096 cm^-1^ (DNA backbone O-P-O stretching). These peaks were the result of a decreased contribution of DNA components in the nucleus, which is consistent with previous studies suggesting that the nucleus is a potential target for DAPT treatment 6,43,44. However, positive peaks related to proteins (1001, 1125, 1256, 1450, 1656 cm^-1^) and lipids (1306, 1450, 1656 cm^-1^) were also observed, whose spectral contribution is increased due to pro-apoptotic proteins responding to DAPT treatment.

PCA analysis *via* PC2 (Figure 5C) shows obvious separation between the DAPT-treated and untreated organelle regions. The PC2 loading is dominated by negative nucleic acid (901 [40], 1339 [40], and 1422 [40] cm^-1^) features probably due to the change in nucleic acid content in the organelle after DAPT treatment. Moreover, the PC1 loading plot showed positive values at 901 cm^-1^ (deoxyribose, (v(CC)_ring_, fatty acids(v(CC), v(CO)) [40], 941 cm^-1^ (C-C BK str.α-helix), 1339 cm^-1^ (G(v(CC)ring)) [40], and 1656 cm^-1^ (amide I, α-spiral and C=C str.).

In Figure 5D, a slight separation could be observed between DAPT-treated and untreated groups. This shows that DAPT also affects the cytoplasm although less so than the nucleus. Meanwhile, the spectral loading of PCs still provides some useful information on the drug response including the increased spectral contribution of proteins and lipids with positive peaks at 747, 1125, 1256, 1306, 1450, and 1656 cm^-1^ in PC1. The score loading of PC2 can be utilized to differentiate the effectiveness induced by two different drug doses in which negative peaks at 1300, 1450 cm^-1^, and 1656 cm^-1^ show a decreased lipid contribution after increasing dose.

In Figure 5E, according to PC1 and the corresponding loading, no obvious differentiation was seen between the untreated cells and the treated cells in the cell membrane region. PC1 exhibited positive values at 1125 cm^-1^ (C-N, C-C stretching), 1256 cm^-1^ (amide III), 1306 cm^-1^ (CH_2_ twist), 1450 cm^-1^ (CH deformation), 1580 cm^-1^ (adenine and guanine), and 1656 cm^-1^ (amide I, α-spiral and C=C str.). The positive loading of PC2 mainly shows the characteristics of protein and lipid (1450, 1656 cm^-1^) in the untreated cell membrane.

## Discussion

The results indicate that different doses of DAPT cause different changes in the Raman spectral feature of OS cells including intensity changes and wavenumber shifts. A visualized understanding of the subcellular effects of an γ-secretase inhibitor was presented through a label-free, non-fixed, *in vitro* cell study using CRMI method. The clustered spectral features depict both the morphology and biochemical changes in the nucleus, cytoplasm, cell organelle, and membrane. Some important cell responses induced by different drug doses were illustrated by a clear representation of inter-group variance in each cellular structure by PCA.

At lower DAPT doses (10 µM), the peak intensities of proteins and nucleic acids increased significantly at 658 cm^-1^ (tyrosine), 1001 cm^-1^ (phenylalanine), 1256 cm^-1^ (amide III) and 1096 cm^-1^ (DNA backbone O-P-O stretching). At higher doses at 20 µM, the peak intensities of most cellular components were significantly reduced. We hypothesized that the protein components are folded in an ordered structure before 10 µM DAPT treatment. The Phe/Tyr side chains may be "buried" or "masked" so that their Raman intensity appears to be rather weak. When treated with anti-tumor drugs like DAPT, hydrogen bonds will be interrupted, and the phenylalanine/tyrosine side chain will be "exposed". Thus, C-H will produce a large amount of Raman scattering at 658, 1001, 1256, and 1450 cm^-1^ 45. At high concentrations (20 µM), cells may have begun to undergo apoptosis, and the peaks of nucleic acids, proteins, and lipids in the cells decrease. The sharp band at 1001 cm^-1^ corresponds to the symmetric circular breathing pattern of phenylalanine and is very sensitive to cell death. This peak can also be used to determine the kinetics of cell apoptosis 46.

**Figure 6:**
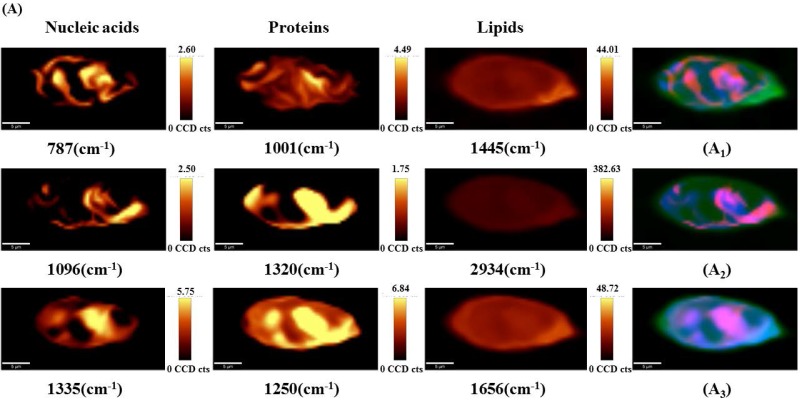

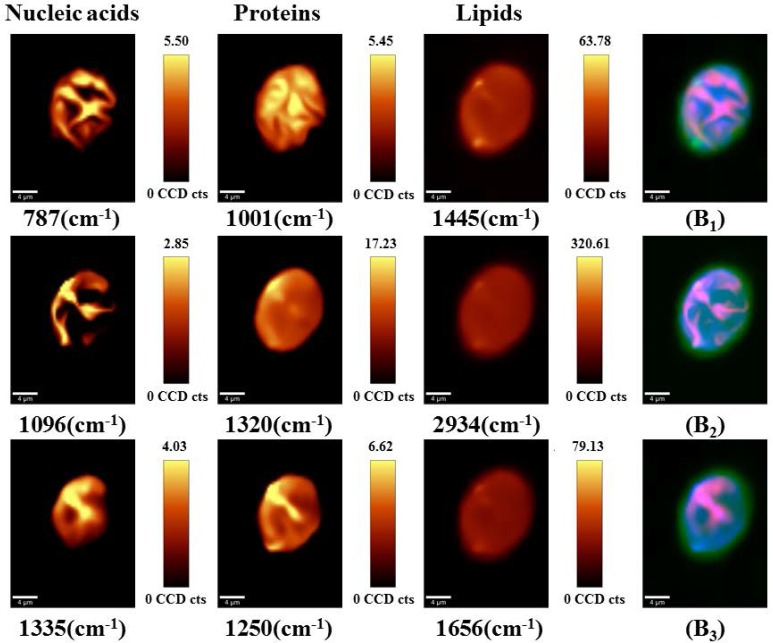

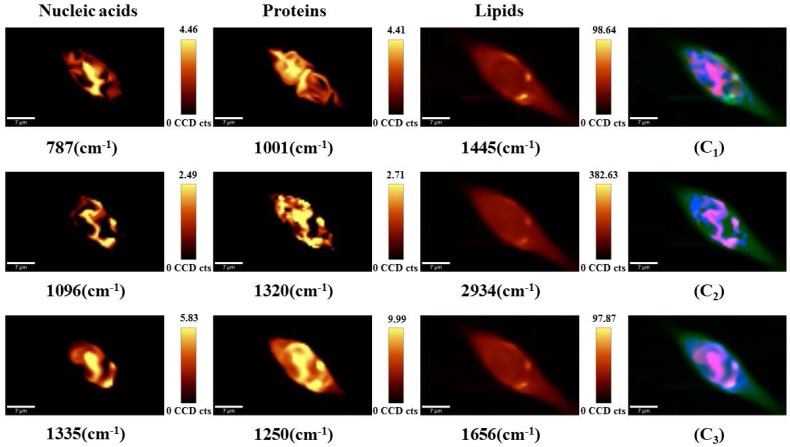
Point-scanned Raman images of nucleic acids (787, 1096, 1335 cm^-1^), proteins (1001, 1250, 1320 cm^-1^) and lipids (1445, 1656, 2934 cm^-1^) in osteosarcoma cells obtained by integration of labeled peaks. (A) untreated cells; (B) 10 μM DAPT-treated cell; (C) 20 μM DAPT-treated cell.

To visualize the drug-induced distribution variations of the main sub-cellular components, univariate spectral imaging was performed at the Raman bands of nucleic acids (787, 1096, 1335 cm^-1^), proteins (1001, 1250, 1320 cm^-1^), and lipids (1445, 1656, 2934 cm^-1^) as shown in Figure 6. For untreated cells (Figure 6A), most of the nucleic acids are concentrated in the nuclear region, and proteins and lipids are distributed throughout the cells. In contrast, the nucleic acids (787, 1335, 1096 cm^-1^) were more dispersed in the nucleus as the drug concentration increased (Figures 6B and C). Proteins (1001, 1250, 1320 cm^-1^) and lipids (1445, 1656, 2934 cm^-1^) were still highly distributed throughout the cells and showed no significant changes. Thus, we hypothesize that changes in the nucleic acid distribution may be due to the cell response of DAPT-induced nuclear fragmentation for triggering apoptosis.

Multivariate analysis presents the entire hyperspectral information of the sample in an unambiguous and uncorrelated manner 47. Although KCA results in a visualized plot of spectrally differentiated regions, it did not provide sufficient spectral information to reveal the biochemical differences among the investigated groups as shown in Figure 3 and 4. Clustered sub-cellular structures can be further analyzed using PCA that provides information about the source of variability in the dataset from underlying molecular changes during cellular-drug interactions 36. The red cluster in Figure 3 (cell membrane) is a hallmark with intense lipids bands at 1125, 1306, 1450, 1656, 2884 and 2934 cm^-1^ (Figure 4A). For these bands, the peak intensity of the lipids increased after treatment with 10 µM DAPT treatment, and the lipids (1125, 1450, 1656, 2884 cm^-1^) decreased after treatment with 20µM DAPT. PCA analysis on the same structure (Figure 5E) showed that the loading spectra of PC1 had similar spectral features of lipids from 1100 to 1700 cm^-1^; however, the untreated and treated cell membranes could not be clearly distinguished in score plots due to the fact that DAPT barely affects the biochemical constitution of the cell membrane. The PCA results of the cytoplasm did not show a clear separation among groups. After drug treatment, the characteristics of the cytoplasm (blue clusters in Figure 3), protein (658, 747, 1001, 1125, 1256, 1450, 1656 cm^-1^), and lipid (1125, 1306, 1450, 1656 cm^-1^) dropped significantly as shown in Figure 4B. The purple cluster in Figure 3 is identified as the nucleus and is a highly aggregated region of nucleic acids (787, 902, 1096, 1335, and 1580 cm^-1^). The intensities of the nucleic acid peaks in the cells reduced, which is consistent with the mechanism of apoptotic cells. In the nuclear region, PCA can distinguish between untreated cells and treated cells. This confirms that the main cellular mechanism of DAPT responses are characterized by alterations in the nuclear constitution and architecture.

## Conclusion

In this work, CRMI was used to analyze the underlying therapeutic mechanism of dose-related osteosarcoma cell responses to DAPT drugs. The drug-induced subcellular compositional changes were firstly analyzed by an inter-group comparison on the spectral features of cells from UT, 10 T, and 20 T groups, which addressed relative spectral contributions of protein, nucleic acids, and lipid content. KCA analysis was then used to visualize the drug-induced subcellular structural changes with a pair-wise comparison of the mean spectra from each clustered region. Based on that, the reconstructed pseudo-hierarchical cluster trees further illustrated some drug induced effects on different cell structures, such as decreased cell viability of cytoplasm, and some subtle content (tyrosine, lipid, nucleic acid) changes in organelle and nuclear. Subsequently, the early apoptotic effects in the nuclear region was explained by PCA with a clear separated scatter plot and decreased spectral contribution of DNA components in nucleus. Furthermore, univariate imaging displayed the distribution pattern of certain cellular components before and after drug treatment. All of these results reported the DAPT-induced cellular changes and heterogeneity at different doses in a label-free manner for understanding drug therapeutic mechanisms and efficacy studies.

## Supplementary Material

Supplementary figure and information.

## Figures and Tables

**Table 1:** Raman peak assignments for osteosarcoma cells.

Raman Shifts (cm^-1^)	Assignment			
	Proteins	Carboh-ydrates	Nucleic acids	Lipids
658	C-C twist, Tyr			
725			A	
787			C,T,U,bk (OPO)	
840		Glucose		
941	C-C BK str.α-helix	C-O-C glycos		
1001	Phe			
1049			RNA v(OPO)	
1096			bk (PO_2_)	
1172	C-H bend Tyr			
1256	Amide III			
1320	CH def			
1450	CH_2_ def			CH def
1580			G, A	
1656	Amide I, α-spiral			C=C str.
2934				CH_3_

Abbreviations: str, stretching; def, deformation; bk, backbone; Tyr, tyrosine; Phe, phenylalanine; A, adenine; T, thymine; C, cytosine; G, guanine; U - uracil.

## Abbreviations

OC: osteosarcoma cell
DAPT: N-[N-(3, 5-difluorophenyl acetyl)-L-alanyl]-sphenylglycine butyl ester
HSD: Honest Significant Difference
UT: Untreated control group
10T: 10 μM DAPT treated
20T: 20 μM DAPT treated
KCA: K-means clustering analysis
PCA: Principal component analysis
OS: osteosarcoma
MTT: 3-4,5-dimethylthiazol-2,5 diphenyl tetrazolium bromide
EMT: epithelial-mesenchymal transition
CRMI: Confocal Raman microspectral imaging
PCs: Principal components
Phe: Phenylalanine
Tyr: Tyrosine
