# Supplementary material for: Confocal Raman Spectral Imaging Study of DAPT, a γ-secretase Inhibitor, Induced Physiological and Biochemical Reponses in Osteosarcoma Cells
Source: Int J Med Sci. 2020 Feb 10;17(5):577–90. doi: 10.7150/ijms.43506 (PMC7085205; doi:10.7150/ijms.43506)
Supplement: Supplementary file 1 — Supplementary figure and information. [file ijmsv17p0577s1.pdf]

-Supporting Information-

Confocal Raman Spectral Imaging Study of DAPT, a γ-secretase Inhibitor, Induced Physiological and Biochemical Reponses in Osteosarcoma Cells

Jie Li[1 #], Rui Wang [2, #], Jie Qin [2, *], Haishan Zeng[3], Kaige Wang[1], Qingli He[4], Difan Wang[5], Shuang Wang[1, *]

[1] Institute of Photonics and Photon-Technology, Northwest University, Xi'an, Shaanxi 710069, China

[2]Department of Orthopedics, The Second Affiliated Hospital of Xi'an Jiaotong University, Xi'an, Shaanxi 710004, China

[3]Imaging Unit – Integrative Oncology Department, BC Cancer Research Center, Vancouver, BC, V5Z1L3, Canada

[4] Department of Physics, Northwest University, Xi'an, Shaanxi 710069, China

[5] School of Life, Xidian University, Xi'an, Shaanxi 710071, China

*Corresponding Author:
Dr. Shuang Wang
Institute of Photonics and Photon-technology,
Northwest University
#229 North Taibai Road
Xi'an, 710069
Shaanxi, China
Email: swang@nwu.edu.cn;
Tel: +86-29-8830-3281
Fax: +86-29-8830-3281

1. The spectral contribution of nucleic acid in cell nucleus

The DAPT induced cellular changes was identified by the morphology and spectral characteristics between untreated cells and treated cells, testified that DAPT mainly acted in the nuclear region. We calculate the content changes in the nucleic acid contribution based on spectra presented in Fig. 4D. It could be found that both 787 and 1096 cm^{-1} bands showed a similar variation patterns of spectral contribution in UT, 10T and 20T groups. For 1096cm^{-1} bands in Nucleus, it showed a same variation pattern with the spectral contribution of 1096cm^{-1} bands by using mean spectra in Fig. 1. However, the spectral contribution of 787 cm-1 bands increased in the 10T group and gradually decreased in 20T group. Since 787 cm^{-1} peak is associated with uracil, thymine, and cytosine ring breathing modes in DNA and RNA bases, its spectral contribution variation may be due to DNA damage and DNA-repair pathway inhibition in DAPT-treated cells, especially in nuclear. The hypnosis is that after 10μM DAPT

action, the cell proliferation would be interrupted, which may prevent cell division at G phase. Thus, the number of chromosomes is increased, and the content of nucleic acid is improved.

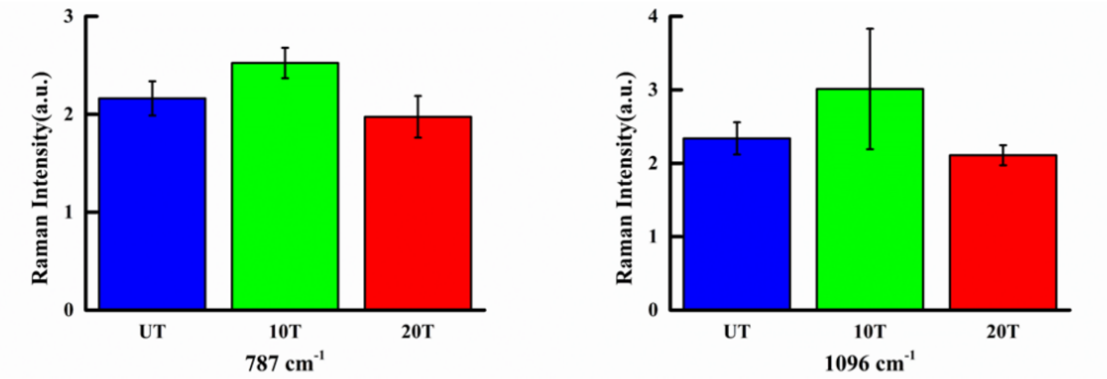

Fig.1S: Box chart displaying the relative spectral contributions of nucleic acid at 787 and 1096 cm^{-1} in the clustered nuclear region of sampled osteosarcoma cells in the UT group (blue), 10 UT group (green) and 20 UT group (red).
